# Berberine Improves Glucose Homeostasis in Streptozotocin-Induced Diabetic Rats in Association with Multiple Factors of Insulin Resistance

**DOI:** 10.5402/2011/519371

**Published:** 2011-11-20

**Authors:** Yanfeng Chen, Yanwen Wang, Junzeng Zhang, Changhao Sun, Alfonso Lopez

**Affiliations:** ^1^National Research Council Canada, Institute for Nutrisciences and Health, Charlottetown, PE, Canada C1A 4P3; ^2^Department of Nutrition and Food Hygiene, Public Health College, Harbin Medical University, Harbin, Heilongjiang 150081, China; ^3^Institute of Public Health Inspection, Heilongjiang Province Center for Disease Control and Prevention, Harbin, Heilongjiang 150036, China; ^4^Department of Pathology and Microbiology, University of Prince Edward Island, Charlottetown, PE, Canada C1A 4P3

## Abstract

The present study was carried out to determine the effect of berberine on glucose homeostasis and several biomarkers associated with insulin sensitivity in male Wistar rats with intraperitoneal injection of streptozotocin (STZ)-induced diabetes. Rats with fasting blood glucose 16.7 mmol/L after 2 weeks of STZ injection were divided into two groups. One group was used as the diabetic control and another treated by gavage feeding with 100 mg/kg/d of berberine in water containing 0.5% carboxymethyl cellulose. A group of rats without receiving STZ was used as the normal control. After 7 weeks, berberine supplementation moderately but significantly lowered fasting blood glucose levels and improved oral glucose tolerance. Berberine lowered plasma free fatty acids and C-reactive protein levels without affecting plasma insulin levels. Diabetic rats treated with berberine showed significantly lower plasma triacylglycerol and cholesterol levels. Furthermore, berberine inhibited dipeptidyl peptidase-4 and protein tyrosine phosphatase-1B activities. In conclusion, berberine showed a dramatic effect of lowering blood cholesterol and triacylglycerols and improved moderately glucose homeostasis in STZ-induced diabetic rats in association with multiple factors related to insulin resistance.

## 1. Introduction

Berberine (BBR) is a medically important isoquinoline alkaloid, which exists in a number of medicinal plants and displays a broad array of pharmacological effects [1]. BBR has been used in Chinese traditional medicine to treat various infectious diseases. Recent years, this natural compound has been increasingly studied for its benefits against various metabolic diseases including diabetes. Several early studies done in streptozotocin- (STZ-) induced diabetic rats demonstrated favorable effects of BBR on blood glucose control [2–4]. A recent study in rats supported the hypoglycemic effect of BBR; however, the efficacy was much less pronounced [5]. Moreover, the mechanism through which BBR improves diabetes is less understood.

Diabetes is a multiple-faceted disease. Increased oxidative stress and chronic inflammation and decreased circulating adiponectin all affect insulin secretion and sensitivity [6, 7]. It is reported that BBR is protective against oxidative stress [8–11]. However, this observation was not supported by the results of a recent study in diabetic rats [5]. The anti-inflammatory effect of BBR has been demonstrated in several *in vitro* studies, showing an inhibitory effect on the gene expression of TNF-*α* and C-reactive protein (CRP) in macrophages and 3T3-L1 adipocytes [12, 13]. Recent *in vitro* studies also showed a significant increase in the expression of adiponectin in 3T3-L1 adipocytes treated with BBR [13, 14]. It remains important to determine whether BBR produces similar effects on chronic inflammation, oxidative stress, and circulating adiponectin levels *in vivo*.

Increases of circulating free fatty acids and triacylglycerols affect not only cardiovascular health but also insulin secretion and function. High levels of blood triacylglycerols are associated with elevated circulating free fatty acids [15], which affects both pancreatic beta cell function [16] and peripheral insulin sensitivity [17]. It is reported that a progressive increase of plasma free fatty acids causes a dose-dependent inhibition of insulin-stimulated glucose uptake and utilization in humans [18]. However, information about how BBR affects circulating free fatty acids, triacylglycerols, and other lipids in diabetes is limited.

The importance of dipeptidyl peptidase-4 (DPP-4) degrades glucagon-like peptide-1, which plays a critical role in insulin secretion and signalling [19]. In general, obese subjects present much higher risk of developing insulin resistance and diabetes. Coincidently, a twofold increase in DPP-4 release is observed in obese subjects compared to the leans and reduced to the lean level after weight loss [20]. Interestingly, plasma DPP-4 activity increases in rats after STZ injection and positively correlates with blood glucose levels [21]. Protein tyrosine phosphatase 1B (PTP-1B) affects insulin signalling through mediating the upstream of phosphatidylinositol 3-kinase pathway and involves mostly the dephosphorylation of insulin receptor and insulin receptor substrates [22]. The importance of these two enzymes in the regulation of glucose homeostasis and insulin signalling has been increasingly recognized.

In the present study, we determined the hypoglycemic and insulin-sensitizing effects of BBR in rats with STZ-induced diabetes. We have further measured blood inflammatory biomarkers, free fatty acids, insulin, adiponectin, lipid profiles, liver oxidative stress biomarkers, as well as DPP-4 and PTP-1B activities, aiming to better understand the mechanisms by which BBR improves glucose metabolism and homeostasis in diabetes.

## 2. Materials and Methods

### 2.1. Animals and Diets

Thirty-eight male Wistar rats (Charles River Laboratories, Montréal, Québec, Canada), 150–170 g, were housed individually in cages in a temperature controlled room with a 12-hour light : dark cycle. Rats were fed a casein-cornstarch-sucrose-based semisynthetic AIN-93G diet containing 5% fat (beef tallow : sunflower oil mix (96 : 4, wt/wt), with free access to diet and water. After 2 weeks of adaptation, rats (*n* = 28) were fasted overnight and then injected with 50 mg/kg body weight of STZ (Sigma Chemicals, Oakville, Ontario, Canada) dissolved in citrate buffer (pH 4.5) to induced diabetes. Normal control animals (NC, *n* = 10) were fasted overnight and injected with the citrate buffer vehicle. Fasting blood glucose in STZ-treated rats was measured 3 days later and a second injection was applied if rats were not diabetic. Fasting blood glucose was measured again 12 days after the first STZ-injection. Rats with fasting blood glucose levels over 16.7 mmol/L were divided into 2 groups (*n* = 10 each) and continued on the AIN-93G diet. These two groups were matched for body weight and blood glucose levels. One group was used as a diabetic control (DB) and the other was orally gavaged with berberine chloride (98% pure, Sigma Chemicals) dissolved in 0.5% carboxymethyl cellulose at a dose of 100 mg/kg/d (BBR). The DB and NC groups were gavaged with the control vehicle of 0.5% carboxymethyl cellulose. During the 7-week treatment period, body weight and food intake were recorded weekly and daily, respectively.

At the end of the study, animals were fasted overnight and anaesthetized with isoflurane (Pharmaceutical Partners of Canada Inc., Richmond Hill, ON). Blood samples were collected from the abdominal aorta into EDTA tubes and placed on ice. After centrifugation, plasma was collected and stored at −80°C. Liver samples were obtained, immediately frozen in liquid nitrogen and stored at −80°C. The animal use and experimental protocols were approved by the Joint Animal Care and Research Ethics Committee of the National Research Council Canada—Institute for Nutrisciences and Health and the University of Prince Edward Island. The study was conducted in accordance with the guidelines of the Canadian Council on Animal Care.

### 2.2. Fasting Blood Glucose Measurements and Oral Glucose Tolerance Testing

Fasting blood glucose was measured at weeks 2, 3, 4, 6, and 7 in lateral tail vein blood samples using an ACCU-Check glucose meter (Roche Diagnostics, Laval, Quebec, Canada). Fasting blood glucose in plasma samples was also measured in triplicate at the beginning and end of the study with a commercial kit (Genzyme, Charlottetown, PE, Canada). Oral glucose tolerance testing was conducted during week 6 of treatment. Following overnight fasting, rats were orally gavaged with 2 g/kg of glucose dissolved in water (40%, wt/v). Blood samples were collected from tail vein at 0, 15, 30, 60, and 120 minutes and measured for glucose levels with commercial kits (Genzyme Diagnostics, Charlottetown, PE, Canada).

### 2.3. Plasma Insulin and Adiponectin Measurement

Plasma insulin and adiponectin levels were quantified in duplicate using commercial ELISA kits from Crystal Chem Inc. (Downer's Grove, IL) according to the manufacturer's instructions. Standards at a series of concentrations were run in parallel with the samples. The concentrations of insulin and adiponectin were calculated in reference to the corresponding standard curves.

### 2.4. Measurement of Liver Oxidative Stress Biomarkers

Liver tissue was homogenized in 250 *μ*L RIPA buffer containing protease inhibitor and centrifuged at 1,600 ×g for 10 minutes at 4°C. The supernatant was collected and measured for superoxide dismutase activity and malondialdehyde concentration according to the kit instructions (Cayman Chemical Company, Ann Arbor, MI). For liver glutathione and oxidized glutathione analyses, homogenized tissue was centrifuged at 10,000 ×g for 15 minutes at 4°C and the supernatant was removed and stored on ice. Protein was removed using MPA (metaphosphoric acid; 5 g MPA in 50 mL dH_2_O) and 4 M TEAM (triethanolamine; 531 *μ*L TEAM in 469 *μ*L dH_2_O) reagents. The deproteinated supernatant was measured for GSH concentrations following the kit instructions. For liver GSSG, GSH in the supernatant was first derivatized with 1 M 2-vinylpyridine (108 *μ*L 2-vinylpyridine in 892 *μ*L ethanol) and then the assay was performed as per GSH.

### 2.5. Measurement of Plasma Inflammatory Cytokines

Plasma TNF-*α* was quantified in duplicate using a commercial ELISA kit (Pierce Endogen, Rockford, IL). To the anti-TNF-*α* antibody-coated 96-well plate, 50 *μ*L of samples or standards were added, followed by one-hour incubation at room temperature. After 3 washes with wash buffer, 50 *μ*L of a biotinylated antibody reagent was introduced and the plate was incubated for 1 hour at room temperature. A second wash cycle was performed followed by addition of 100 *μ*L of substrate TMB (3,3′,5,5′-tetramethylbenzidine) and a 30-minutes incubation in a dark room. The reaction was terminated by adding 100 *μ*L of stop solution and absorbance was read at 450 nm. TNF-*α* concentrations were calculated in reference to the standard curve. Plasma CRP was measured in duplicate with a rat high-sensitivity CRP ELISA kit (Kamiya Biomedical Company, Seattle, WA) following the kit instructions.

### 2.6. Plasma Lipids and Free Fatty Acid Analyses

Plasma total cholesterol, HDL-cholesterol, and triacylglycerols were measured in duplicate using a Pointe-180 chemistry analyzer (Pointe Scientific Inc., Canton, MI), with all reagents purchased from the same company. Non-HDL cholesterol was calculated by subtracting HDL-cholesterol from total cholesterol. Plasma nonesterified fatty acids concentrations were measured in triplicate with a commercial kit (Wako Diagnostics, Richmond, VA).

### 2.7. Inhibition of DPP-4 and PTP-1B by BBR

Effect of BBR on the activity of DPP-4 and PTP-1B was measured in triplicate using the commercial kits (Enzo Life Sciences, Plymouth Meeting, PA) following the manufacturer's instructions. IC50 was obtained for each enzyme with a series dilution of BBR in the assay buffer.

### 2.8. Statistical Analysis

Data analyses were performed by one-way ANOVA using SAS 9.1 (SAS Institute, North Carolina, USA). Plasma HDL-cholesterol, non-HDL-cholesterol, and triacylglycerols were logarithmically transformed before statistical analysis. Differences between treatment means were determined by pairwise comparisons using the least squares means test, where *P* < 0.05 was considered significant. Results are presented as mean values with their standard errors.

## 3. Results

### 3.1. BBR Had No Effect on Food Intake and Body Weight in Diabetic Rats

All rats in the normal control group completed the study. Two rats in the DB and three rats in the BBR groups either died or were euthanized due to dehydration and hyperglycemia during weeks 5 to 7 of the treatment. In comparison to normal controls, diabetic rats displayed increased (*P* < 0.0001) food intake by over 60% during each week of the 7-week treatment period (Table 1). BBR did not show any effect on food intake. Diabetes decreased weight gain, resulting in significantly lower (*P* < 0.0001) body weights in diabetic rats compared to normal control rats after 2 weeks of STZ-injection and over the rest of the treatment period (Table 1). BBR treatment did not improve weekly body weights during the entire treatment period. Body weights in DB and BBR groups were 90% of the normal control at the beginning and decreased to 56% of the normal control at the end of treatment. 

### 3.2. BBR Improved Fasting Blood Glucose Levels and Oral Glucose Tolerance

**Figure 1:**
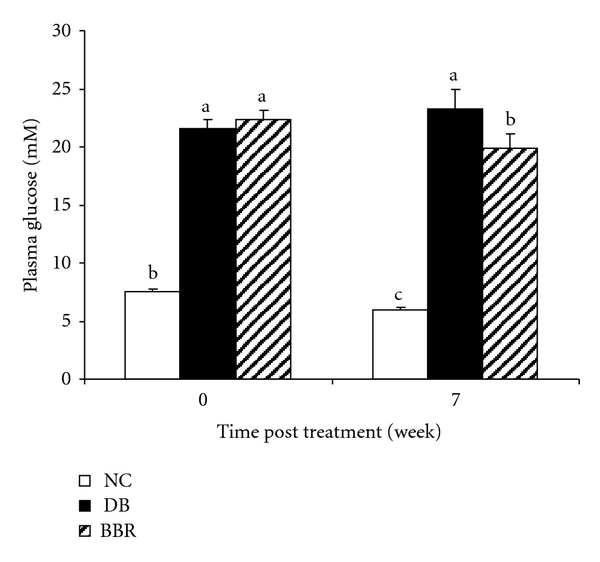
Oral administration of BBR lowered fasting blood glucose levels in streptozotocin-induced diabetic rats. NC: normal control; DB: diabetic control; BBR: diabetic rats gavaged with 100 mg/kg/d of berberine chloride dissolved in 0.5% carboxymethyl cellulose. The DB and NC groups were gavaged with the control vehicle of 0.5% carboxymethyl cellulose. Values are means ± S.E.M. (at week 0, *n* = 10 for all groups; at week 7, *n* = 10, 8, and 7 for the NC, DB, and BBR, resp.). a, b, c Mean values with different letters were significantly different (*P* < 0.05).

Fasting blood glucose was measured weekly with a glucose meter. At week 3, rats in the BBR group showed significantly lower (*P* < 0.05) fasting blood glucose levels than diabetic rats (25.3 ± 1.7 mmol/L in BBR-treated rats versus 29.0 ± 1.5 mmol/L in diabetic controls). At the end of the study, fasting plasma glucose levels were measured in plasma using a commercial glucose kit. The results show that rats treated with BBR exhibited decreased fasting blood glucose levels by 20% (*P* < 0.01) as compared with the diabetic control (Figure 1).

**Figure 2:**
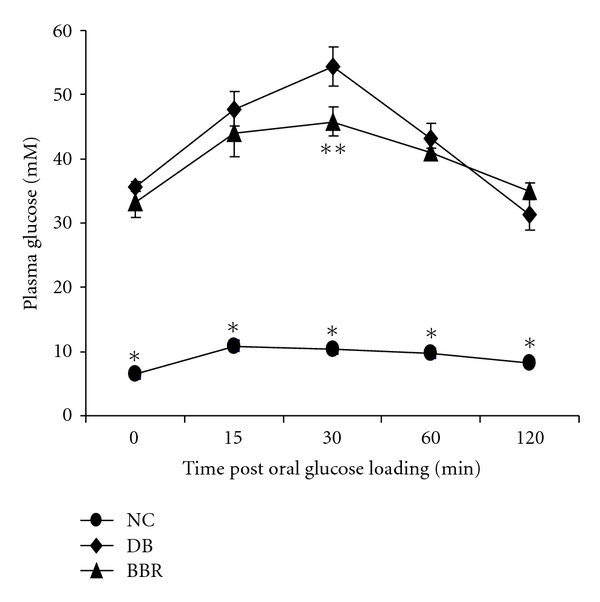
BBR treatment improved oral glucose tolerance in streptozotocin-induced diabetic rats. NC: normal control; DB: diabetic control; BBR: diabetic rats gavaged with 100 mg/kg/d of berberine chloride dissolved in 0.5% carboxymethyl cellulose. The DB and NC groups were gavaged with the control vehicle of 0.5% carboxymethyl cellulose. Values are means ± S.E.M. (*n* = 10, 8, and 7 for the NC, DB, and BBR, resp.). *different from the DB and BBR groups (*P* < 0.0001), respectively. **different from the DB group (*P* < 0.05).

Oral glucose tolerance test was performed during week 6 of the treatment. Diabetic rats showed impaired glucose tolerance, which was improved following BBR treatment (Figure 2). At 30 minutes post oral glucose loading, blood glucose was significantly lower (*P* < 0.05) in rats treated with BBR relative to diabetic controls.

### 3.3. Effect of BBR on Plasma Levels of Insulin, Adiponectin, and Inflammatory Biomarkers

As expected, STZ injection dramatically decreased circulating levels of insulin. At the beginning of treatment, diabetic rats maintained approximately 22% of blood insulin as compared to normal controls (2.34 ± 0.41 in the normal control versus 0.52 ± 0.10 in the diabetic group). After 7 weeks, plasma insulin levels decreased to 8% of the normal control in the diabetic group (*P* < 0.0001) and were not improved in the BBR group (10% of the normal control, Table 2). It is suggested that BBR treatment did not recover insulin secretion function of pancreatic *β*-cells.

Blood CRP concentration is an important biomarker of chronic inflammation. The analysis in the current study revealed a tendency of increase (*P* = 0.09) of plasma CRP in diabetic rats (Table 2). After 7 weeks of treatment with BBR, plasma CRP levels were reversed (*P* < 0.05) and became similar with the normal control. The plasma levels of adiponectin and TNF-*α* were not significantly affected by BBR (Table 2).

### 3.4. BBR Improved Plasma Lipid and Free Fatty Acid Profiles

In the present study, we found that plasma total cholesterol, non-HDL-cholesterol, and triacylglycerols were significantly increased (*P* < 0.05) in STZ-induced diabetic rats but decreased (*P* < 0.05) after BBR treatment (Table 3). Diabetic rats showed almost onefold increase (*P* < 0.05) of plasma-free fatty acids over normal controls. After BBR treatment, plasma-free fatty acids were lowered (*P* < 0.05) to the normal control levels.

### 3.5. BBR Had No Effect on Liver Antioxidant Status in STZ-Induced Diabetic Rats

To determine whether antioxidation is involved in the antidiabetic properties of BBR, we measured liver malondialdehyde, glutathione and oxidized glutathione levels, and superoxide dismutase activity. The analysis did not indicate any significant changes of these parameters in diabetic rats treated with BBR (data not shown).

### 3.6. BBR Inhibited the Activity of DPP-4 and PTP-1B

**Figure 3:**
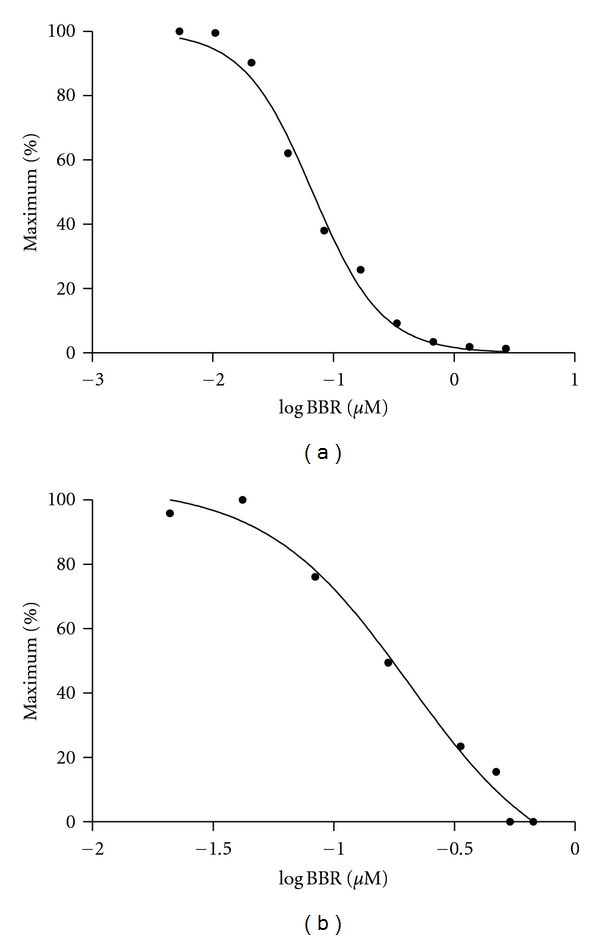
Dose response of BBR on DPP-4 (a) and PTP-1B (b) enzyme activities.

To determine whether DP-4 and PTP-1B were involved in the beneficial effects of BBR on diabetes and glucose metabolism, the activity of these two enzymes was measured in the presence of different concentrations of BBR. It was found that BBR inhibited DPP-4 and PTP-1B activities in a dose-dependent manner. The IC50 for DPP-4 and PTP-1B was 67 *μ*M and 205 *μ*M, respectively (Figure 3).

## 4. Discussion

Free fatty acids are an important causative factor of insulin resistance and diabetes. Plasma-free fatty acids are elevated in most obese subjects who also display a significantly higher rate of diabetes. Similarly, increasing blood-free fatty acids inhibits insulin-stimulated glucose uptake into muscle [23]. This muscle insulin resistance is caused by a free fatty acid-induced defect in insulin-stimulated glucose transport or phosphorylation, or both. This insulin resistance is also caused by a second defect consisting of inhibited glycogen synthase activity [23]. The increase of free fatty acid flux stimulates hepatic gluconeogenesis while inhibiting glycogen synthesis and thus induces hepatic insulin resistance [24, 25]. In addition, a constantly high concentration of free fatty acids in the bloodstream increases the accumulation of triacylglycerols in both liver and muscle. Because triacylglycerols are in a state of constant turnover, increase of triacylglycerols results in a local accumulation of triacylglycerol metabolites including acyl-coenzyme A, ceramides, and diacylglycerols. These metabolites can activate a serine kinase cascade, leading to defects in insulin signaling and glucose transport [26]. Moreover, the modulation of transcription by free fatty acids through their binding to peroxisome proliferator-activated receptors impairs glucose metabolism [26]. This consequence of events is frequently referred to as lipotoxicity [27]. In the present study, diabetic rats showed significantly higher levels of blood-free fatty acids, which were reversed by oral administration of BBR. The reduction of blood-free fatty acids was possibly achieved through increasing fat oxidation by upregulating AMP-activated protein kinase [28]. It is suggested that BBR improves glucose homeostasis in diabetic rats at least in part through lowering circulating free fatty acids.

Apart from elevated circulating free fatty acids, chronic inflammation is also strongly linked with diabetes [29, 30] and insulin resistance [31]. Inflammation causes insulin resistance via inhibiting the signaling downstream of insulin receptor. Exposure of murine adipocytes [32] or hamster ovary cells [33] to TNF-*α* stimulates phosphorylation of serine residues on insulin receptor substrate-1 (IRS-1). This phosphorylation reduces subsequent tyrosine phosphorylation of IRS-1 in response to insulin and its ability to associate with the insulin receptor, thereby inhibiting downstream insulin signaling [32, 34, 35]. The significant reduction of plasma CRP levels in diabetic rats following BBR treatment indicates that inhibition of chronic inflammation is another contributory factor, in addition to the decrease of free fatty acids, to the observed improvement of fasting blood glucose levels and oral glucose tolerance in BBR-treated diabetes rats. The anti-inflammatory effect of BBR has been reported in several *in vitro* studies. A study in rat mesangial cells showed that BBR inhibits the activation of nuclear factor *κ*B signaling and the protein expression of its downstream inflammatory mediators [36]. BBR reduces the gene expression of inflammatory biomarker CRP in 3T3-L1 adipocytes [13] and several other inflammatory biomarkers such as TNF-*α* in macrophages [12]. On the other hand, the anti-inflammatory effect of BBR could be a second (or confounding) effect of free fatty acid reduction as there is a strong association between blood-free fatty acids and chronic inflammation in both obese [37] and normal, healthy subjects [15]. Free fatty acids evoke inflammation via activating the proinflammatory nuclear factor *κ*B pathway [38], and BBR decreased free fatty acids.

An additional benefit of BBR on diabetes derives from its favorable effect on plasma lipids. In comparison with normal rats, diabetic rats showed significantly higher levels of plasma total cholesterol, non-HDL-cholesterol, and triacylglycerols. After BBR treatment, plasma concentrations of these lipid parameters were markedly lowered. In particular, BBR decreased plasma triacylglycerol levels, consistent with the decrease of plasma-free fatty acids. As dyslipidemia is one of the important components of diabetes and insulin resistance [39, 40], the reductions of blood lipids, in particular triacylglycerols by BBR, are beneficial to insulin sensitivity and glucose utilization in the peripheral tissues [26].

DPP-4 and PTP-1B play important roles in glucose metabolism [19, 22]. The increased activity of these two enzymes is associated with the development of insulin resistance and diabetes. BBR inhibited the activity of these two enzymes in a dose-dependent manner, suggesting that suppression of DPP-4 and PTP-1B activity was involved in the insulin-sensitizing effect of BBR.

In addition to insulin sensitivity, insulin insufficiency is another important causative factor of diabetes. However, BBR was not able to promote the pancreas to regenerate *β* cells or restore *β* cell function in rats of STZ-induced diabetes, in agreement with our previous study [5]. This corroborates data observed in other animal models such as db/db mice and diet-induced diabetic mice [41]. As reported previously [5], BBR did not show any antioxidant effect in diabetic rats.

In conclusion, BBR supplementation lowered fasting blood glucose and improved oral glucose tolerance in rats with intraperitoneal injection of STZ-induced diabetes. BBR decreased plasma chronic inflammation biomarker CRP, circulating free fatty acids, and triacylglycerols levels and inhibited DPP-4 and PTP-1B activities. The results collectively demonstrate that BBR lowers considerably the blood cholesterol and triacylglycerols while moderately improving insulin sensitivity in association with multiple risk factors of insulin resistance and diabetes.

## Figures and Tables

**Table 1:** Effect of berberine on food intake and body weight in STZ-induced diabetic rats.

			*Food intake *(g/d)					
Treatment		Week 1	Week 2	Week 3	Week 4	Week 5	Week 6	Week 7
NC		23.9 ± 1.1^b^	24.7 ± 1.8^b^	24.7 ± 1.6^b^	24.4 ± 1.5^b^	22.2 ± 2.2^b^	23.1 ± 1.8^b^	27.2 ± 2.3^b^
DB		45.3 ± 1.1^a^	41.2 ± 1.8^a^	40.9 ± 1.7^a^	39.3 ± 1.6^a^	38.1 ± 2.3^a^	41.1 ± 1.9^a^	44.0 ± 2.4^a^
BBR		43.7 ± 1.2^a^	43.6 ± 1.9^a^	42.2 ± 1.8^a^	40.9 ± 1.7^a^	36.6 ± 2.4^a^	43.3 ± 2.1^a^	43.6 ± 2.6^a^

			*Body weight *(g)					
Treatment	Week 0	Week 1	Week 2	Week 3	Week 4	Week 5	Week 6	Week 7

NC	277.4 ± 3.1^a^	316.4 ± 6.7^a^	356.7 ± 9.4^a^	385.5 ± 10.9^a^	417.0 ± 13.0^a^	439.0 ± 14.4^a^	457.5 ± 14.9^a^	483.6 ± 15.9^a^
DB	248.4 ± 5.7^b^	258.9 ± 7.5^b^	269.4 ± 10.5^b^	260.5 ± 12.1^b^	268.5 ± 14.5^b^	265.9 ± 16.1^b^	267.8 ± 16.7^b^	272.2 ± 18.7^b^
BBR	249.0 ± 5.3^b^	266.3 ± 8.0^b^	275.3 ± 11.2^b^	274.1 ± 13.0^b^	276.0 ± 15.5^b^	261.6 ± 17.2^b^	272.3 ± 17.8^b^	272.7 ± 19.0^b^

NC: normal control; DB: diabetic control; BBR: diabetic rats gavaged with 100 mg/kg/d of berberine chloride dissolved in 0.5% carboxymethyl cellulose. The DB and NC groups were gavaged with the control vehicle of 0.5% carboxymethyl cellulose. Data are means ± S.E.M. (*n* = 10 for all groups from week 0 to 5; *n* = 10, 9 and 8 at weeks 5 and 6 for the NC, DB, and BBR, resp.; and *n* = 10, 8, and 7 at week 7 for the NC, DB, and BBR, resp.).

^a,b^For each week, mean values bearing unlike superscript letters were different, *P* < 0.05.

**Table 2:** Effect of berberine on plasma insulin, adiponectin, and CRP levels in rats with STZ-induced diabetes.

Treatment	Insulin (pmol/L)	Adiponectin (*μ*g/mL)	TNF-*α* (pg/mL)	CRP (*μ*g/mL)
NC	453.5 ± 61.8^a^	5.21 ± 0.4^a^	33.2 ± 3.4^a^	199.1 ± 10.0^ab^
DB	29.2 ± 6.9^b^	6.49 ± 0.36^a^	26.7 ± 3.4^a^	224.9 ± 8.6^a^
BBR	36.1 ± 9.0^b^	6.70 ± 0.61^a^	36.0 ± 9.8^a^	191.6 ± 11.3^b^

NC: normal control; DB: diabetic control; BBR: diabetic rats gavaged with 100 mg/kg/d of berberine chloride dissolved in 0.5% carboxymethyl cellulose. The DB and NC groups were gavaged with the control vehicle of 0.5% carboxymethyl cellulose.

CRP: C-reactive protein.

Data are means ± S.E.M. (*n* = 10, 8, and 7 for the NC, DB, and BBR, resp.).

^a,b^For each parameter, values bearing unlike superscript letters were different, *P* < 0.05.

**Table 3:** Effect of berberine on plasma lipids and free fatty acids in STZ-induced diabetic rats.

Treatment	T-C (mmol/L)	HDL-C (mmol/L)	non-HDL-C (mmol/L)	TAG (mmol/L)	FFA (mmol/L)
NC	2.12 ± 0.08^c^	1.51 ± 0.06^a^	0.61 ± 0.05^c^	0.99 ± 0.12^b^	0.58 ± 0.11^b^
DB	7.57 ± 1.91^a^	2.18 ± 0.14^a^	5.38 ± 1.98^a^	7.49 ± 2.84^a^	1.11 ± 0.09^a^
BBR	3.81 ± 0.42^b^	2.03 ± 0.19^a^	1.78 ± 0.30^b^	2.01 ± 0.39^b^	0.54 ± 0.02^b^

NC: normal control; DB: diabetic control; BBR: diabetic rats gavaged with 100 mg/kg/d of berberine chloride dissolved in 0.5% carboxymethyl cellulose. The DB and NC groups were gavaged with the control vehicle of 0.5% carboxymethyl cellulose.

T-C: total cholesterol; HDL-C: high-density lipoprotein cholesterol; non-HDL-C: non HDL cholesterol (very low density lipoprotein + intermediate density lipoprotein + low density lipoprotein cholesterol); TAG: triacylglycerols; FFA: free fatty acids.

Data are means ± S.E.M. (*n* = 10, 8, and 7 for the NC, DB, and BBR, resp.).

^a,b,c^For each parameter, mean values bearing unlike superscript letters were different, *P* < 0.05.
